# *Hyalomma dromedarii* infesting camels in Hail Province, Saudi Arabia, carry antimicrobial resistant bacteria

**DOI:** 10.3389/fvets.2025.1662637

**Published:** 2025-10-14

**Authors:** Alanoud T. Aljasham, Sajith Raghunandanan, Raed Farzan, Abdulhadi M. Abdulwahed, Embalil Mathachan Aneesh, Sumiyaa Alharbi, Yusra Shukri, Mohammed Alshammari, Fuad Alanazi

**Affiliations:** ^1^Department of Clinical Laboratory Sciences, College of Applied Medical Sciences, King Saud University, Riyadh, Saudi Arabia; ^2^Department of Public Health and Genomics, Manipal School of Life Sciences, Manipal Academy of Higher Education, Manipal, Karnataka, India; ^3^Centre for Research in Emerging Tropical Diseases (CRET-D), Department of Zoology, University of Calicut, Malappuram, Kerala, India; ^4^Laboratory and Blood Bank Department, Ad-Diriyah General Hospital, Riyadh, Saudi Arabia

**Keywords:** *Hyalomma dromedarii*, antibacterial resistance, zoonotic pathogens, camel tick, Hail province

## Abstract

Ticks are known vectors of various pathogens and are increasingly recognized as carriers of antimicrobial-resistant (AMR) bacteria. However, the role of camel ticks in AMR transmission remains poorly understood. In this study, we investigated bacteria isolated from *Hyalomma dromedarii* hard ticks collected from dromedary camels in Hail Province, Saudi Arabia, and assessed their AMR profiles. A total of 57 ticks were collected, yielding 29 bacterial isolates. The majority (79%; 23/29) were Gram-negative bacteria, primarily *Enterobacter cloacae* complex (*n* = 21) and *Pseudomonas putida* (*n* = 2). Gram-positive isolates (21%; 6/29) included *Staphylococcus sciuri* (*n* = 4) and *Staphylococcus xylosus* (*n* = 2). All Gram-negative isolates were resistant to cefazolin, 91% to amoxicillin/clavulanic acid, and 8.7% to trimethoprim/sulfamethoxazole, while remaining susceptible to higher-generation cephalosporins, carbapenems, and aminoglycosides. Among Gram-positive isolates, resistance to fusidic acid was universal, with occasional resistance to benzylpenicillin (33%) and erythromycin (17%). No multidrug resistance across three or more antibiotic classes was observed. These findings highlight the presence of clinically relevant AMR bacteria in camel ticks and underscore the need for targeted AMR surveillance in arid livestock regions. Such efforts are critical to understanding and mitigating AMR risks within the animal–human–environment interface of the One Health framework.

## Introduction

Antimicrobial resistance (AMR) has emerged as a significant global public health challenge (1–3). The rise of AMR has made many conventional antibiotic therapies ineffective, necessitating combination antibiotic regimens. This, in turn, contributes to the emergence of multidrug-resistant (MDR) bacteria, perpetuating a vicious cycle of resistance development (4). Global modeling for 2019 indicates that antimicrobial resistance was implicated in about 4.95 million deaths worldwide, including 1.27 million deaths directly caused by drug-resistant bacterial infections (5). AMR bacteria have been identified in humans, animals, food, plants, and various environmental compartments, including water, soil, and air (6, 7). These resistant organisms are frequently transmitted from their natural reservoirs to humans and animals, facilitating cross-species transmission (8, 9).

Although numerous studies have investigated various modes of AMR transmission within the environment (10–13). There is limited literature available on the transmission of AMR through arthropod vectors (14, 15). Ticks are well-established vectors of various pathogens affecting both humans and animals, and they represent a significant threat to public and veterinary health (16, 17). Humans can be infected with various tick-borne bacterial diseases, including human granulocytic anaplasmosis caused by *Anaplasma phagocytophilum*, Lyme disease, spotted fever caused by Spotted fever group rickettsiae, Q-fever caused by *Coxiella burnetti*, and Tularemia caused by *Francisella tularensis* (18–20). The composition of the tick microbiome is complex, shaped by interactions between symbiotic bacteria, the host, and the surrounding environment (21). During blood-feeding, ticks can transmit pathogens and microorganisms and may facilitate the transfer of AMRs between hosts (22). Given their broad geographic distribution and ability to parasitize a wide range of hosts, including domestic animals, wildlife, migratory birds, and pets, ticks are increasingly recognized as potential reservoirs and vectors for AMR dissemination (23, 24). Despite this, most existing research has focused primarily on tick-borne pathogens, often overlooking the potential public health risks associated with AMR transmission (14). Furthermore, the effects of tick species, parasitic versus free-living life stages, and intergenerational transmission on the distribution and persistence of AMR remain poorly understood.

The present study aims to investigate the prevalence, diversity, and potential AMR determinants of bacteria harbored by ticks infesting camels, a major livestock species in Saudi Arabia. Despite the growing importance of camels in regional food security and veterinary health, the role of camel-associated ticks as reservoirs and vectors of AMR bacteria remains poorly understood. By identifying tick species, isolating bacterial communities, and characterizing antimicrobial resistance patterns, this research aims to characterize the bacterial communities and antimicrobial resistance profiles carried by camel ticks, thereby providing a baseline for future studies focused on transmission. The findings will contribute to national surveillance systems, inform antimicrobial stewardship, and support targeted interventions within the One Health framework. Ultimately, this study aims to strengthen public and animal health, promote environmental sustainability, and protect vital natural resources.

## Materials and methods

### Selection of study area

**Figure 1:**
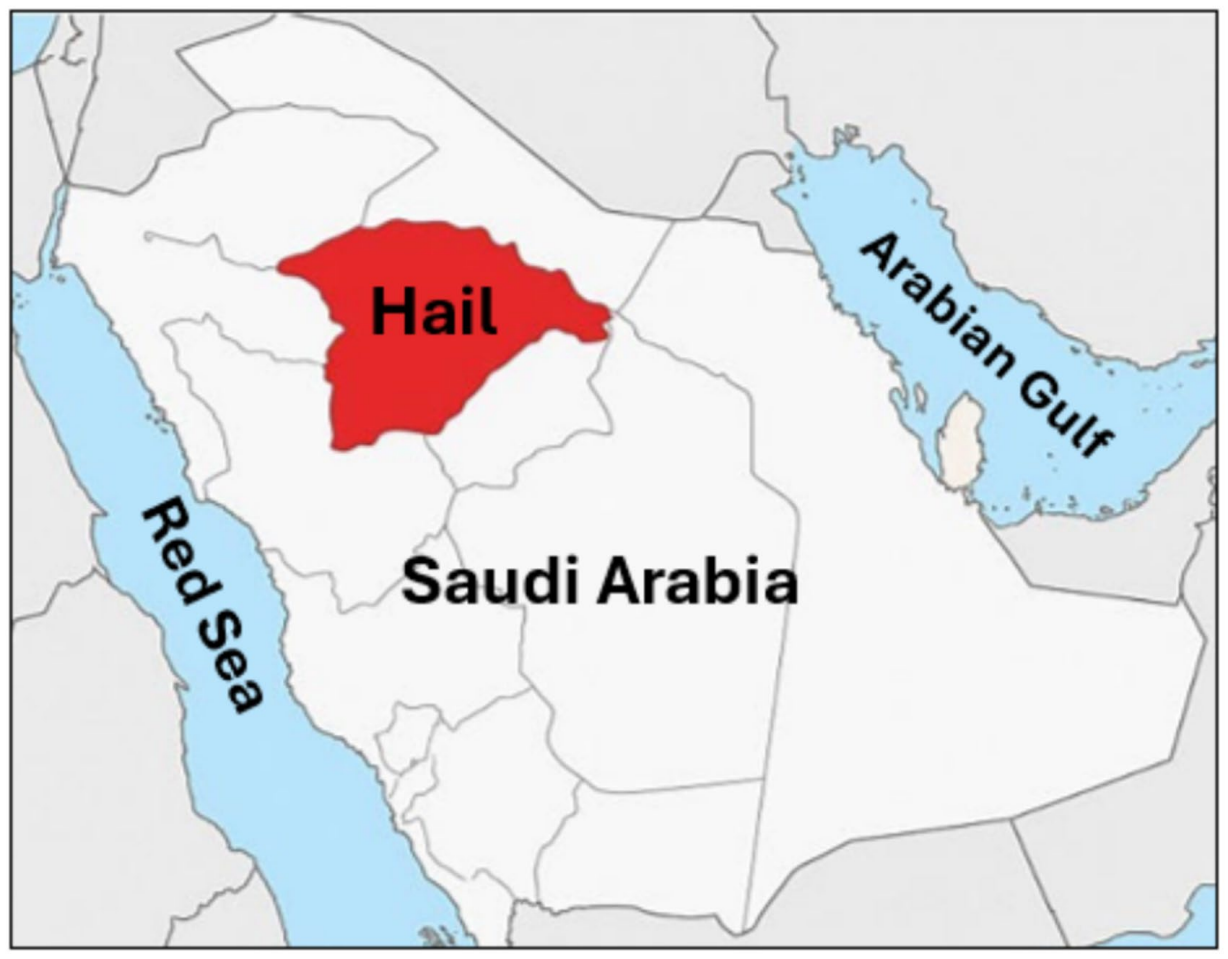
Geographic location of the study area. Map illustrating the Hail Province (highlighted in red) in the northern region of Saudi Arabia, where tick specimens were collected from dromedary camels.

This research was done in Hail City, located in the northwestern region of Saudi Arabia (Figure 1). Hail is characterized by its unique topography, including mountains, deserts, and fertile agricultural areas, supported by a semi-arid climate with moderate rainfall compared to other regions of the Arabian Peninsula. These environmental conditions foster crop cultivation and livestock farming, making Hail a vital hub for agricultural and pastoral activities. The region’s substantial camel population and frequent human-animal interactions provided an ideal setting for investigating tick-borne bacterial pathogens.

### Collection and identification of ticks

A total of 57 ticks were collected from multiple sites within a 20-mile radius of Hail City, Saudi Arabia (27.42476° N, 41.73580° E) during the months of October and November. Specimens were manually obtained from 13 female dromedary camels at the Hail Camels’ Market by targeting preferred attachment sites such as the perianal region, udder, and inner thighs. Each specimen was carefully detached using fine-tipped forceps and immediately placed into a sterile 20 mL plastic container with a perforated lid to ensure adequate ventilation.

Post-collection, ticks were maintained under controlled conditions with relative humidity ranging from 70 to 93% and temperature between 20 and 26 °C to preserve their viability. Viability assessments were conducted upon processing, and notable biological observations were recorded, such as egg-laying in engorged females.

Morphological identification was carried out using a stereomicroscope to examine structural features in detail, employing taxonomic keys based on external morphology, developmental stage, and sex-specific traits (25). Further species confirmation was achieved through molecular identification targeting the mitochondrial 16S rRNA gene. Genomic DNA was extracted using the Qiagen DNeasy Blood & Tissue Kit, following the manufacturer’s instructions. A partial fragment of the 16S rRNA gene was amplified using primers described by Black and Piesman (26): Forward 5′-CTGCTCAATGATTTTTAAATTGCTGTGG-3′ and Reverse 5′-CCGGTCTGAACTCAGATCAAGT-3′. PCR amplification was performed in our laboratory in 20 μL reaction volumes, using thermal cycling conditions adapted from Mangold et al.^27^, with minor modifications: an initial denaturation at 94 °C for 3 min, followed by 35 cycles of 94 °C for 30 s, 55 °C for 30 s, and 72 °C for 1 min, concluding with a final extension at 72 °C for 5 min. PCR products were visualized using 2% agarose gel electrophoresis and purified with a commercial gel extraction kit. The purified amplicons were submitted to Macrogen, Inc. (Seoul, Republic of Korea) for bidirectional sequencing.

### Bacterial isolation from ticks

Ticks collected from camels were processed through multiple steps to ensure effective bacterial isolation. Initially, each tick was surface-sterilized by immersion in 70% ethanol for 5 min, followed by three sequential washes with sterile phosphate-buffered saline (PBS) to eliminate external contaminants. Internal tissues were then aseptically extracted. Homogenization of tick tissues was performed under aseptic conditions inside a Class II biosafety cabinet (Thermo Fisher Scientific). Sterile disposable gloves, laboratory coats, and face masks were used throughout the procedure. All instruments were sterilized prior to use, and sterile PBS was employed. Tick tissues were manually homogenized using autoclaved glass homogenizers; although a tissue homogenizer machine could reduce human contact, the manual method was adopted due to equipment constraints while ensuring sterility at all times. The resulting homogenate was transferred into a nutrient broth (Sigmaaldrich, Germany) for enrichment and incubated at 37 °C incubator (Thermo Fisher Scientific, US) with constant agitation at 250 rpm for 24 h. Following enrichment, samples were cultured on blood agar and MacConkey agar plates to facilitate the growth of a broad spectrum of bacterial species. All media and broths, including nutrient broth, blood agar (Oxoid, United Kingdom), and MacConkey agar (Himedia, United States), were prepared according to standard microbiological protocols (27). Media were sterilized by autoclaving at 121 °C and 15 psi for 15 min before use. Blood agar plates were prepared by supplementing sterilized base agar with 5% defibrinated sheep blood under aseptic conditions. Following the culturing, plates were incubated for 24 h at 37 °C. Next day, colonies were selected based on morphology and pigmentation. Finally, bacterial isolates were preserved at −80 °C in glycerol stocks for further analysis.

### Identification of bacterial isolates

For bacterial identification, the isolated microorganisms were identified using the Gram staining technique to distinguish between Gram-positive and Gram-negative bacteria. Following Gram staining, bioMérieux Vitek 2 Compact System was used for both identification and antimicrobial sensitivity testing. The BioMérieux Vitek 2 Compact System with Gram-positive (GP) ID REF21342 and Gram-negative (GN) ID REF21341 cards were used according to manufacturer guidelines.

### Antimicrobial susceptibility testing

Antimicrobial susceptibility testing was performed on 29 bacterial isolates using the Vitek 2 Compact System. Through specialized cards, AST-N417 for GN and AST-P580 for GP, this system determines the minimum inhibitory concentration (MIC) for various antibiotics against the bacteria. The antibiotics tested represented multiple classes for both Gram-positive and Gram-negative bacteria. The antibiotics used for Gram-positive bacteria are benzylpenicillin, oxacillin, gentamicin, tobramycin, levofloxacin, moxifloxacin, erythromycin, clindamycin, linezolid, teicoplanin, vancomycin, tetracycline, tigecycline, nitrofurantoin, fusidic acid, rifampicin, and trimethoprim/sulfamethoxazole. For Gram-negative bacteria, the antibiotics used are amoxicillin / clavulanic acid, piperacillin/tazobactam, cefazolin, cefuroxime, cefuroxime axetil, ceftazidime, ceftriaxone, cefepime, ertapenem, imipenem, meropenem, amikacin, gentamicin, ciprofloxacin, fosfomycin, nitrofurantoin, and trimethoprim/sulfamethoxazole. After analysis, the MIC cutoff values were used to distinguish each isolated bacterium that may be sensitive, intermediate, or antibiotic-resistant. The results were issued using Vitek 2 compact software.

## Results

### Collection and identification of the hard ticks from the hail province, Saudi Arabia

**Figure 2:**
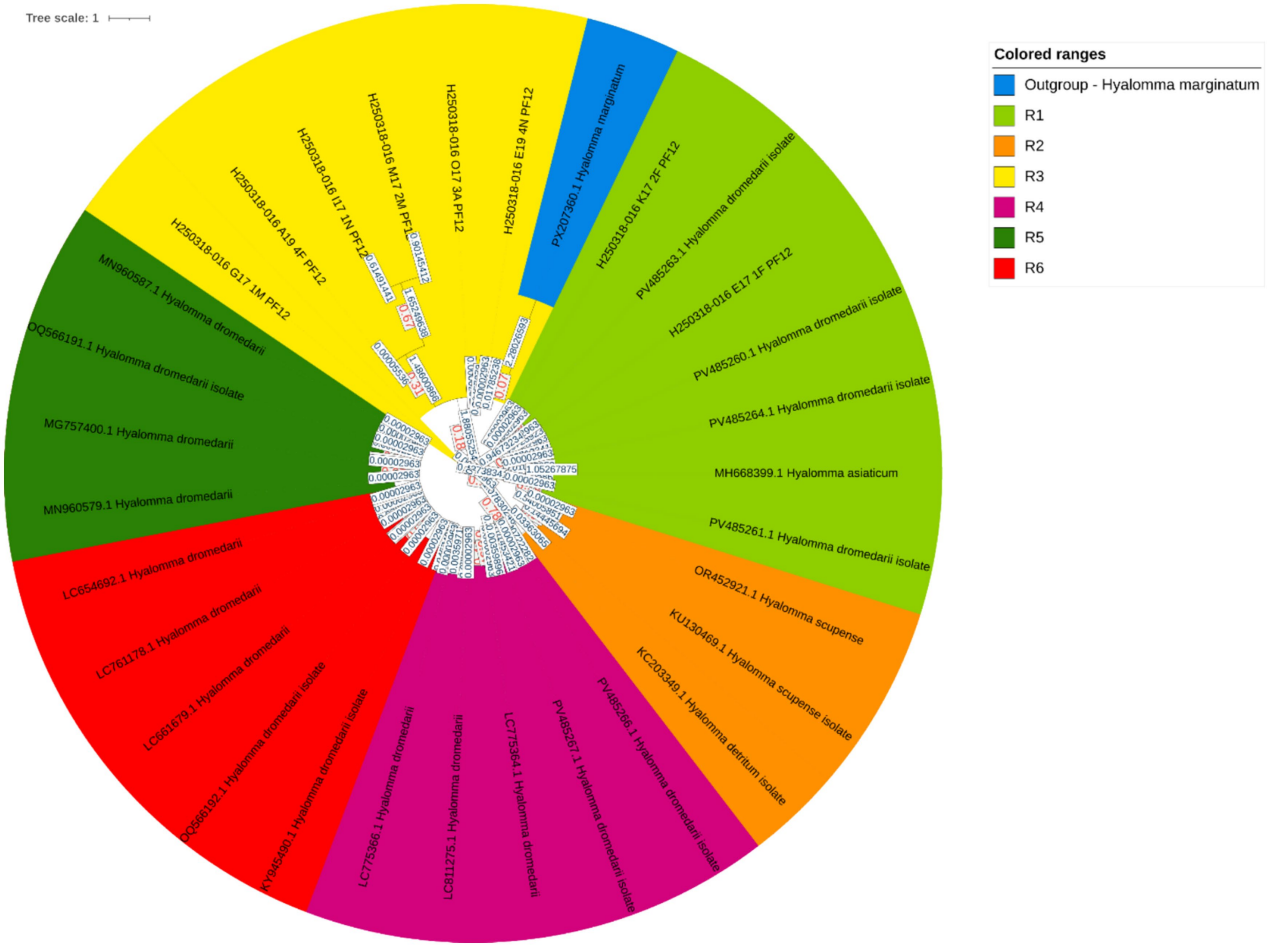
Evolutionary analysis by the Maximum Likelihood method. The evolutionary history was inferred by using the Maximum Likelihood method and the Tamura-Nei model (51). The tree with the highest log likelihood −3500.14 is shown. Initial tree(s) for the heuristic search were obtained automatically by applying Neighbor-Join and BioNJ algorithms to a matrix of pairwise distances estimated using the Tamura-Nei model and then selecting the topology with the superior log likelihood value. The tree is drawn to scale, with branch lengths measured in the number of substitutions per site. This analysis involved 31 nucleotide sequences. Of these, 6 sequences were generated in this study (GenBank accession numbers: PV485260, PV485261, PV485263, PV485264, PV485266, PV485267), while the remaining 25 sequences were retrieved from GenBank. Codon positions included were 1^st^ + 2^nd^ + 3^rd^ + Noncoding. There was a total of 286 positions in the final dataset. The phylogenetic tree comprises seven distinct clades, color-coded as follows: Clade R1 (green), Clade R2 (light green), Clade R3 (yellow), Clade R4 (yellow-orange), Clade R5 (orange), Clade R6 (dark orange), and Clade R7 (red), each representing a separate evolutionary lineage. The following software was used for phylogenetic tree construction: iTOL version 7.2 (https://itol.embl.de/tree/15258218106142911744696289).

A total of 57 ticks were collected from 13 female dromedary camels within Hail Province, Saudi Arabia. The relatively low tick burden observed during this period is likely attributable to the moderately cool climatic conditions, as higher tick densities are typically associated with the warmer summer months. The collected ticks were categorized by developmental stage and engorgement status into three groups: non-engorged adults (*n* = 18, 31.6%), engorged adults (*n* = 28, 49.1%), and nymphs (*n* = 11, 19.3%). All ticks remained viable until the time of experimentation, and oviposition was observed in several engorged females. To confirm species identity, molecular characterization was performed by targeting the mitochondrial 16S rRNA gene. The sequences generated in this study have been deposited in GenBank under accession numbers PV485260–PV485267. The results identified all specimens as *Hyalomma dromedarii*, and their evolutionary relationships are depicted in Figure 2.

### Identification of the isolated bacteria from the ticks

**Figure 3:**
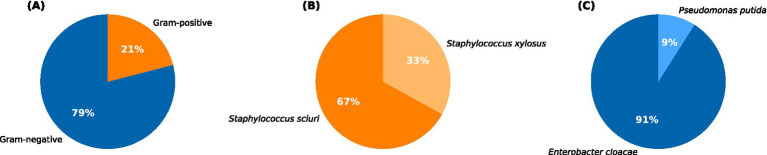
Pie chart showing: **(A)** the percentage distribution of Gram-positive and Gram-negative bacteria identified from the total tick population. The percentage distribution of major types of Gram-positive and Gram-negative bacteria is represented in **(B,C)**, respectively.

From the collected ticks, employing various microbial culturing techniques, a total of 29 bacterial species were isolated. The isolated bacteria were later subjected to Gram staining, followed by strain identification using the Vitek 2 Compact system. The results revealed that the bacterial isolates belonged to four different species. Of these, approximately 79% (*n* = 23) were Gram-negative bacteria, and 21% (*n* = 6) were Gram-positive bacteria (Figure 3A). The high-throughput detection identified the prevalent Gram-positive bacteria as *Staphylococcus sciuri* (*n* = 4), followed by *Staphylococcus xylosus* (*n* = 2; Figure 3B) which are reported potential pathogens to humans. Among the Gram-negative bacteria, the most common species was *Enterobacter cloacae* complex (*n* = 21), followed by *Pseudomonas putida* (*n* = 2; Figure 3C).

### Determining the antimicrobial susceptibility of the isolated bacteria

**Figure 4:**
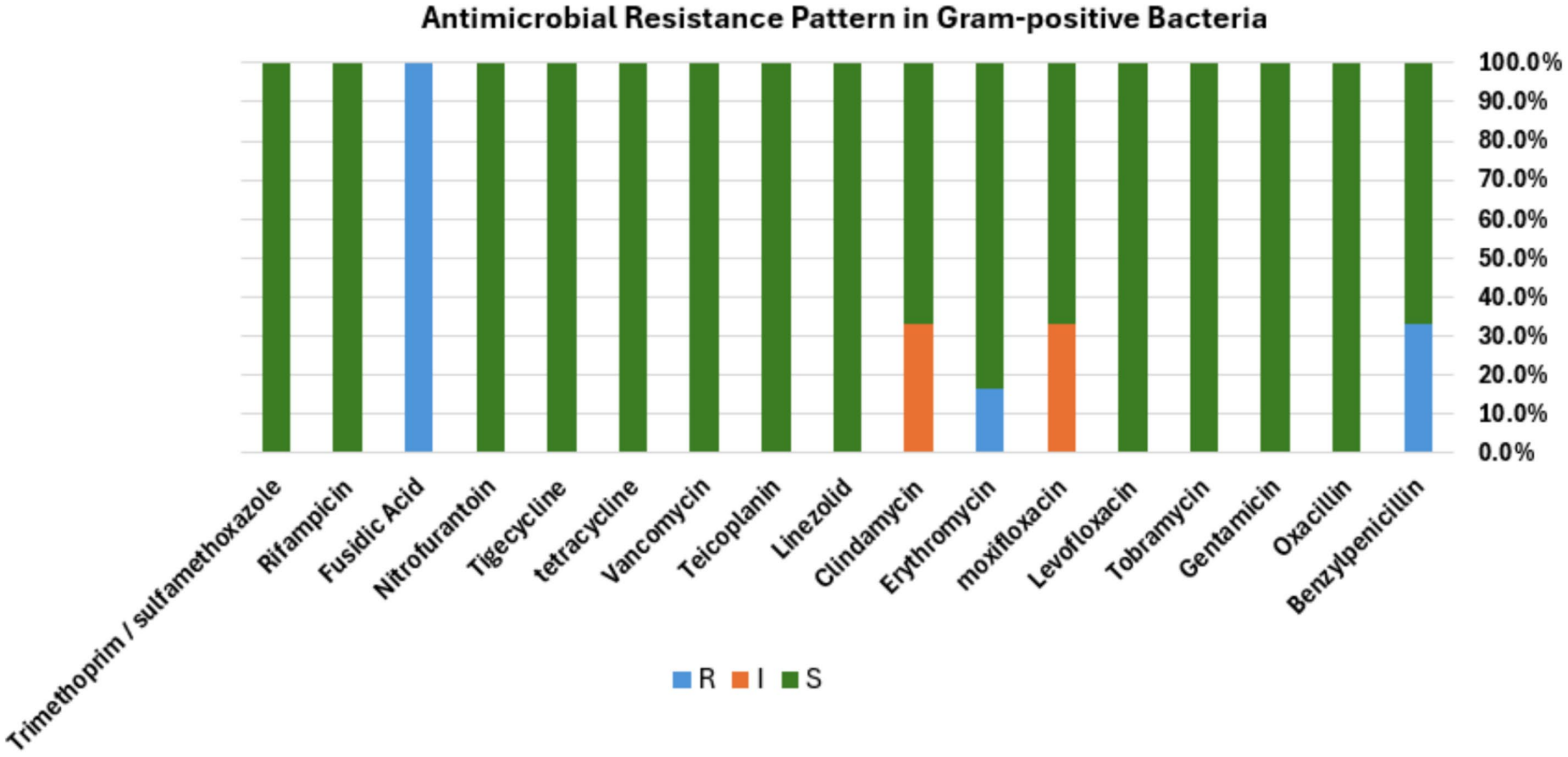
Antibacterial susceptibility pattern. Bar diagram representing the antibiotic susceptibility profile of Gram-positive bacterial isolates tested against a panel of commonly used antibiotics. The bar shows the percentage of isolates classified as resistant (R, blue), intermediate (I, orange), or susceptible (S, green) to each antibiotic, respectively.

**Figure 5:**
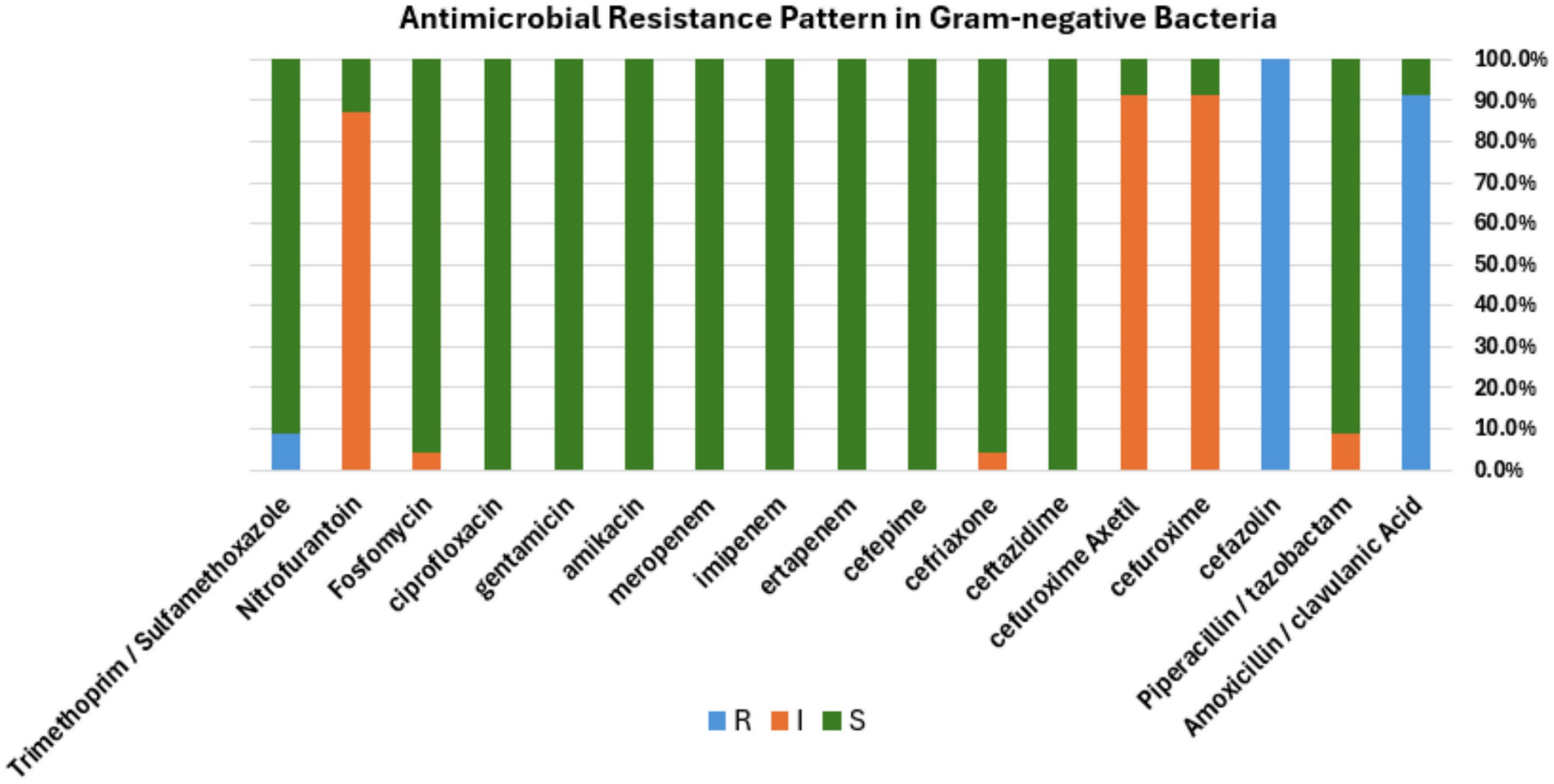
Antibacterial susceptibility pattern. Bar diagram representing the antibiotic susceptibility profile of Gram-negative bacterial isolates tested against a panel of commonly used antibiotics. The bar shows the percentage of isolates classified as resistant (R, blue), intermediate (I, orange), or susceptible (S, green) to each antibiotic, respectively.

To identify the drug susceptibility pattern of these bacterial isolates, all 29 bacterial isolates were tested for antimicrobial susceptibility using the Vitek 2 compact system. The result showed that both Gram-positive and Gram-negative bacteria exhibited resistance to several antimicrobial agents (Figures 4, 5). The antibacterial suitability test for the Gram-positive bacteria was as follows: *For Staphylococcus sciuri*, 100% (*n* = 4/4) were resistant to fusidic acid, 33.3% (*n* = 2/4) were resistant to benzylpenicillin, and 16.7% (*n* = 1/4) were resistant to erythromycin. About 50% (*n* = 2/4) of this isolate showed intermediate resistance to moxifloxacin and clindamycin. 100% (*n* = 4/4) of *Staphylococcus sciuri* were sensitive to oxacillin, gentamicin, tobramycin, levofloxacin, linezolid, teicoplanin, vancomycin, tetracycline, tigecycline, nitrofurantoin, rifampicin, and trimethoprim/sulfamethoxazole. The other species*, Staphylococcus xylosus,* showed 100% (*n* = 2/2) resistance to fusidic acid and 100% (*n* = 2/2) sensitivity to oxacillin, gentamicin, tobramycin, levofloxacin, linezolid, teicoplanin, vancomycin, tetracycline, tigecycline, nitrofurantoin, rifampicin, and trimethoprim/sulfamethoxazole (Figure 4).

For the Gram-negative bacteria, antimicrobial agent sensitivity testing was as follows: *Enterobacter cloacae complex* showed 100% (*n* = 21/21) resistance to amoxicillin /clavulanic acid and cefazolin. This isolate displayed intermediate resistance to three antibiotics, including 100% (*n* = 21/21) to cefuroxime and cefuroxime axetil, 95% (*n* = 20/21) to nitrofurantoin, and 4.7% (1/21) to fosfomycin. About 100% (21/21) of this isolate were sensitive to piperacillin/tazobactam, ceftazidime, ceftriaxone, cefepime, ertapenem, imipenem, meropenem, amikacin, gentamicin, ciprofloxacin, and trimethoprim/sulfamethoxazole. The other species, *Pseudomonas putida*, showed 100% (*n* = 2/2) resistance to cefazolin and trimethoprim/sulfamethoxazole, 100% (*n* = 2/2) intermediate resistance to piperacillin/tazobactam, and 50% (*n* = 1/2) intermediate resistance to ceftriaxone. 100% (*n* = 2/2) of this isolate were sensitive to other antimicrobial agents includes amoxicillin / clavulanic acid, cefuroxime, cefuroxime axetil ceftazidime, cefepime, ertapenem, imipenem, meropenem, amikacin, gentamicin, ciprofloxacin, fosfomycin, and nitrofurantoin (Figure 5). The antibiotic susceptibility of each bacterium highlighted in the present study is represented in Tables 1, 2.

**Table 1:** Antimicrobial susceptibility/resistance patterns of different Gram-positive bacteria isolated from ticks.

No.	Bacteria	Benzylpenicillin	Oxacillin	Gentamicin	Tobramycin	Levofloxacin	Moxifloxacin	Erythromycin	Clindamycin	Linezolid	Teicoplanin	Vancomycin	Tetracycline	Tigecycline	Nitrofurantoin	Fusidic Acid	Rifampicin	Trimethoprim / Sulfamethoxazole
1	*S. sciuri*																	
2	*S. xylosus*																	
3	*S. xylosus*																	
4	*S. sciuri*																	
5	*S. sciuri*																	
6	*S. sciuri*																	

Green represents Sensitive (S), Yellow represents Intermediate (I), and Red represents Resistant (R) isolates, respectively.

**Table 2:** Antimicrobial susceptibility/resistance patterns of different Gram-negative bacteria isolated from ticks.

Lab ID	Bacteria	Amoxicillin / Clavulanic Acid	Piperacillin / Tazobactam	Cefazolin	Cefuroxime	Cefuroxime Axetil	Ceftazidime	Ceftriaxone	Cefepime	Ertapenem	Imipenem	Meropenem	Amikacin	Gentamicin	Ciprofloxacin	Fosfomycin	Nitrofurantoin	Trimethoprim / Sulfamethoxazole
1	*E. cloacae* complex																	
2	*E. cloacae* complex																	
3	*E. cloacae* complex																	
4	*E. cloacae* complex																	
5	*E. cloacae* complex																	
6	*P. putida*																	
7	*E. cloacae* complex																	
8	*E. cloacae* complex																	
9	*E. cloacae* complex																	
10	*E. cloacae* complex																	
11	*E. cloacae* complex																	
12	*E. cloacae* complex																	
13	*E. cloacae* complex																	
14	*E. cloacae* complex																	
15	*E. cloacae* complex																	
16	*E. cloacae* complex																	
17	*E. cloacae* complex																	
18	*E. cloacae* complex																	
19	*P. putida*																	
20	*E. cloacae* complex																	
21	*E. cloacae* complex																	
22	*E. cloacae* complex																	
23	*E. cloacae* complex																	

Colors indicate susceptibility status: green (Sensitive, S), yellow (Intermediate, I), and red (Resistant, R).

## Discussion

The issue of AMR is increasingly affecting human health, driven by the widespread use of antibiotics in clinical settings and agriculture, as well as the accumulation of antibiotics in the environment (28, 29). Ticks, known vectors of various zoonotic diseases, harbor a complex and diverse microbiota. Multiple factors, including tick species, geographic location, environmental conditions, engorgement status, and life stage, can influence the composition of this microbiota (30). However, the role of ticks as potential reservoirs of AMR is an emerging area of research (14). In this research, we investigated the microbial diversity of camel ticks collected across Hail Province, Saudi Arabia, and reported the presence of antimicrobial-resistant bacteria within them.

The present study identified several clinically relevant bacterial species from camel ticks, including *Enterobacter cloacae* complex, *Staphylococcus sciuri*, *Staphylococcus xylosus*, and *Pseudomonas putida*. While not traditionally classified as tick-borne pathogens, some of these species can be associated with opportunistic infections in both humans and animals (31–35). For instance, *Enterobacter cloacae* are frequently reported in hospital-acquired infections and are known to harbor multiple resistance mechanisms (36, 37). *Staphylococcus sciuri* has been isolated from wound and urinary tract infections and is recognized as a reservoir of antimicrobial resistance genes (38, 39). The detection of these species in *Hyalomma dromedarii* suggests that camel ticks may act as incidental carriers of environmental or opportunistic pathogens. This raises important questions regarding the acquisition and potential dissemination of antimicrobial-resistant bacteria within tick populations.

Although it remains unclear whether these bacteria are permanent members of the tick microbiota or transient contaminants acquired from the environment or host, similar genera have been reported in previous studies (25, 40). Environmental conditions, geographical location, and host exposure are likely to influence the microbial communities associated with ticks (41–45). It is plausible that the observed resistance patterns may reflect indirect exposure to antibiotics administered for wound management in camels; however, the absence of formal veterinary treatment records in this study limits the ability to draw definitive conclusions.

As such, the true bacterial diversity harbored by *Hyalomma dromedarii* may be broader than captured in this study. Additionally, the choice of Hail Province as the study area may have influenced the observed microbial composition, given its semi-arid climate, high camel density, and frequent human–animal interactions. These ecological and anthropogenic factors could contribute to the environmental acquisition and potential dissemination of antimicrobial-resistant bacteria by ticks. Future studies employing high-throughput approaches such as 16S rRNA amplicon sequencing or metagenomics will be required to capture the broader microbial diversity of tick populations.

A previous study conducted in the Al-Jouf province of Saudi Arabia profiled the microbiota of camel ticks and reported the predominance of *Staphylococcus lentus*, *Staphylococcus pseudintermedius*, and *Sphingomonas paucimobilis* (25). In contrast, our study did not detect these bacterial species in the tick samples analyzed. Interestingly, we observed a notable prevalence of the *Enterobacter cloacae* complex, which may be attributed to geographical variation influencing microbial diversity. Furthermore, unlike the earlier study, we did not identify any bacterial isolates exhibiting complete resistance to the bactericidal action of benzylpenicillin, oxacillin, clindamycin, or vancomycin. Intermediate resistance phenotypes, such as those observed for *Enterobacter cloacae* against cefuroxime, may reflect intrinsic variability and are not necessarily predictive of clinical treatment failure. Such findings should therefore be interpreted with caution when considering their epidemiological and clinical relevance.

We believe this discrepancy may be explained by differences in geographical topology and the timing of sample collection, which are known to affect the distribution of antimicrobial-resistant bacteria (14). Moreover, several studies on tick microbiomes from different regions have demonstrated that the geographical location of the ticks plays a crucial role in shaping their microbiome (44, 45). Alternatively, studies have suggested that blood-feeding altered the diversity of the tick microbiome and could reduce microbial abundance in ticks (46). Therefore, we cannot exclude the possibility that the lifecycle and feeding stages of the tick under study could contribute to discrepancies in the observed microbial diversity.

Collectively, our study highlights the complex dynamics of antimicrobial resistance mediated by ticks. While we identified the presence of antimicrobial-resistant bacterial pathogens within tick populations across the Hail province of Saudi Arabia, the underlying mechanisms driving this resistance remain unclear. Antibiotic resistance genes (ARGs) are increasingly recognized as emerging environmental contaminants due to their potential for horizontal transfer between bacterial species and across ecosystems, thereby contributing to the emergence and dissemination of multidrug-resistant bacteria (47, 48). We hypothesize, therefore, that these resistant bacteria harbor ARGs that confer antimicrobial resistance, although their specific identities and roles have yet to be elucidated. Furthermore, it is plausible that ARGs are transmitted either vertically through genetic inheritance or horizontally via mobilizable genetic elements (MGEs), facilitating the spread of resistance traits among bacterial populations (49, 50).

### Limitations

This study has several limitations. First, while antimicrobial resistance phenotypes were characterized, the presence or mobility of specific resistance genes was not confirmed at the molecular level and should be investigated in future studies. Second, reliance on culture-based methods may have selectively favored fast-growing species and overlooked fastidious or intracellular bacteria such as *Rickettsia*, *Coxiella*, and *Anaplasma*. This reliance likely led to an incomplete representation of the tick microbiome. Future studies should therefore incorporate high-throughput methods such as 16S rRNA gene sequencing or metagenomic approaches to capture a broader and more comprehensive microbial profile. Third, the geographical focus on Hail Province may limit the generalizability of the findings, as bacterial diversity and resistance profiles can vary regionally. Finally, the cross-sectional design precludes conclusions about temporal dynamics of resistance. Addressing these limitations in future research will provide deeper insights into the role of ticks as reservoirs and disseminators of antimicrobial-resistant bacteria.

## Data Availability

The datasets presented in this study can be found in online repositories. The names of the repository/repositories and accession number(s) can be found at: https://figshare.com/, https://doi.org/10.6084/m9.figshare.30171484, https://www.ncbi.nlm.nih.gov/genbank/, PV485260, https://www.ncbi.nlm.nih.gov/genbank/, PV485261, https://www.ncbi.nlm.nih.gov/genbank/, PV485262, https://www.ncbi.nlm.nih.gov/genbank/, PV485263, https://www.ncbi.nlm.nih.gov/genbank/, PV485264, https://www.ncbi.nlm.nih.gov/genbank/, PV485265, https://www.ncbi.nlm.nih.gov/genbank/, PV485266, https://www.ncbi.nlm.nih.gov/genbank/, PV485267.
